# Fast screening of total nutrient contents in strawberry leaves and spent growing media using NIRS

**DOI:** 10.3389/fpls.2023.1210791

**Published:** 2023-08-21

**Authors:** Bart Vandecasteele, Chris Van Waes

**Affiliations:** Plant Sciences Unit, Flanders Research Institute for Agriculture, Fisheries and Food (ILVO), Melle, Belgium

**Keywords:** crop nutrient uptake, sustainable growing media, peat replacement, controlled environment agriculture (CEA), circular horticulture, high throughput, vertical farming

## Abstract

**Introduction:**

In closed-loop soilless cultivation, the main nutrient sinks are nutrients retained either by the crop or in spent growing media. Measurement of nutrients in spent growing media and in the aboveground vegetative plant biomass at crop termination can be a tool for assessing and optimizing nutrient efficiency. The first aim of this study was to test the potential of near-infrared reflectance spectroscopy (NIRS) to forecast the various nutrient contents in strawberry leaves, which would then allow for assessment of crop nutrient status and total nutrient uptake by strawberry plants. The second aim was to test NIRS as a high throughput technique for assessing the N, K, Ca, Mg and organic matter (OM) content and the pH, EC and C:N and C:P ratios for a dataset of composts, plant fibers and spent growing media. The NIRS prediction model for fast screening of the total nutrient contents in spent growing media was compared with a single extraction method.

**Methods:**

A database with 369 dried and ground strawberry leaf samples with known contents of N, P, K, Ca, and Mg were scanned using NIRS. The database covered a range of leaf contents of 6-35 g N/kg dry matter (DM), 0.7-6.3 g P/kg DM and 2-29 g K/kg DM. A dataset of 458 samples of different types of materials used in growing media was validated with a dataset of 109 samples.

**Results:**

Validation for the strawberry leaves indicated potential for this application, with R^2^ values of 0.90 or higher for N, K and Ca, and R^2^ values higher than 0.85 for P and Mg. Validation for the dataset of composts, plant fibers and spent growing media also indicated the potential for this application, with R^2^ values of 0.90 or higher for organic matter, and with R^2^ values of 0.85 or higher for total Ca, pH and C:N. A first test indicated potential for the calibration based on fresh samples of compost, plant fiber as well as spent growing media or dried (not ground) samples.

**Discussion:**

Use of NIRS on fresh samples would eliminate the need for drying and grinding the samples and would reduce screening time. The ammonium acetate extraction is a reliable alternative to NIRS for fast screening of the total P, K, Ca, and Mg contents in composts, plant fibers and spent growing media.

## 1 Introduction

Soilless cultivation in greenhouses is an example of a controlled environment agriculture system with high nutrient and water use efficiency. In greenhouse cultivation based on closed fertigation systems, two pools of nutrients can be removed after strawberry cultivation: the nutrients taken up in the aboveground plant biomass and the accumulated nutrients in the spent growing media (Vandecasteele et al., 2023). Assessment of these nutrient pools at crop termination is relevant for various applications. This knowledge is useful for both scientific and operational purposes, and indicates whether measures are needed to minimize the occurrence of nutrient sinks (Vandecasteele et al., 2020). For greenhouse management, this assessment can be relevant to measures aiming at increased the nutrient efficiency, including the need for adapted fertigation, and for optimal processing and reuse of the biomass (van Tuyll et al., 2022). Knowledge of the nutrient content of spent growing media is important in light of the possible reuse or recycling of spent media and the enclosed nutrients (Vandecasteele et al., 2020).

Nutrient contents in strawberry leaves at the end of the cultivation may indicate whether any elements were deficient and which of them, if any, actually limited strawberry growth and yield (e.g., Pritts et al., 2015; De Tender et al., 2021). Nutrient content may also interact with diseases and pathogens, thus yielding additional insights into crop optimization *via* the nutrient supply. Renewable materials are increasingly used in growing media blends, which heightens the risk for unbalanced fertigation, as the materials themselves may be a source of nutrients. For this reason, assessment of nutrient contents in virgin growing media at the start of the cultivation is becoming more relevant. Concerns about sustainability of peat extraction are influencing the composition of growing media for professional soilless cultivation, with increasing substitution of peat by other materials (Atzori et al., 2021). The materials currently used in growing media can be clustered into two groups: plant fibers including peat, coir, bark, wood fibers and straw fibers, and composts including green compost, vegetable, fruit and garden waste (VFG) compost and other compost types or digestates (Atzori et al., 2021). Processed spent growing media may be reused in a subsequent cultivation (Vandecasteele et al., 2020). Non-peat materials may contain more nutrients than virgin peat, resulting in a need for an adapted fertilizer application when changing from mainly peat-based growing media to peat reduced blends.

Chemical analyses of leaves and growing media are time-consuming and expensive. In contrast, near infrared reflectance spectroscopy (NIRS) is a sensitive, fast and non-destructive analytical technique and may be a potential solution for fast and cheap chemical and biochemical characterization of materials (Galvez-Sola et al., 2010a; Peltre et al., 2011; Viaene et al., 2017). NIRS has been reported as a fast method of screening of chemical and biochemical properties during the composting process (Galvez-Sola et al., 2010b; Viaene et al., 2017), screening of chemical composition of compost or digestate products (Galvez-Sola et al., 2010a; Peltre et al., 2011; Zennaro et al., 2022) and determination of humic acids in composts (Meissl et al., 2008). NIRS has potential for predicting chemical parameters and phytotoxicity of peats and peat-based growing media (Ludwig et al., 2006; Terhoeven-Urselmans et al., 2008) as well as for determining the degree of maturity and biological stability of composts (Lohr et al., 2016; Erhart et al., 2017). Erhart et al. (2017) used a NIRS model for predicting single maturity parameters and an integrated maturity index based on the dissolved organic carbon, oxygen uptake rate, Solvita maturity index and the nitrate content of composts. Adapa et al. (2011) and Elakiya and Arulmozhiselvan (2021) demonstrated the success of Fourier transform infrared spectroscopy (FTIR) analysis for quantifying lignin, hemicellulose and/or cellulose functional groups in untreated or fiberized cereal straw and coconut coir, respectively, and Higashikawa et al. (2014) used FTIR for predicting the maturity of organic wastes, composts and compost-based growing media.

Literature indicates great potential for application of NIRS for *in situ* nutrient analysis of plant leaf tissue (Ulissi et al., 2011; Lohr et al., 2016; Prananto et al., 2020), and thus for crop monitoring during cultivation. As nutrient contents in the leaves indicate nutrient availability and may reflect deficiency or imbalances, such knowledge may allow growers to discriminate between effects of nutrients versus effects of other factors to optimize crop performance. NIRS can be used for global monitoring/screening of the cultivation, as it is a fast and clean methodology that allows producers to assess crop and fruit quality, nutrient contents and general crop health as well as to identify optimal harvest times, classify the strawberries in a sorting line in real time, and identify the authenticity of strawberry production methods (Amodio et al., 2017). NIRS may be used for monitoring different N forms and N availability in growing media during cultivation (Zhu and Li, 2013).

Another technique for fast screening is based on single extraction methods to assess either nutrient availability or the total nutrient contents in growing media (Handreck, 1995; Hauck et al., 2022). Several sensor-based techniques are available for assessing and managing the N status of the crop based on optical properties (Muñoz-Huerta et al., 2013). For assessing other nutrients than N as well, total contents in selected leaves or other crop parts are determined based on total analysis, or nutrients are assessed based on petiole sap measurements (Bottoms et al., 2013). When signs of unbalanced nutrient uptake by crops are detected, it may be already too late to correct the fertilizer addition in due time. A more proactive approach is soilless cultivation is to assess and optimize the nutrient status in the root zone or the growing medium. This may be achieved based on regular analysis of root solution composition during cultivation (Voogt and Bar-Yosef, 2019), or through chemical single extraction methods (Blok et al., 2019). The nutrients in the root zone or in the extracts are not necessarily correlated to the total nutrient contents in the growing medium: these methods aim at investigating nutrient contents available to plants, and whether the growing medium contains high concentrations of elements which may be damaging to the plant (Blok et al., 2019). Extraction protocols differ in the pH conditions mimicked during extraction, ranging between non-buffered conditions (sample pH is maintained during extraction) and strongly standardized pH conditions (irrespective of the sample pH). In the present study, the ammonium acetate (AmAc) extraction was tested as a single extraction. This method has been developed to assess the need to supply fertilizers to peat-based growing media, but has now been validated for new materials in growing media (composts, plant fibers) and reused growing media.

### 1.1 Aims and objectives

One objective of this study was to assess whether NIRS can be used to measure the total nutrient contents in the aboveground plant biomass of strawberry plants. Another was to assess whether a NIRS calibration model for chemical composition of composts, plant fibers and spent growing media could be used to assess the pH, EC, nutrient and organic matter (OM) content of spent growing media. A third objective was to compare the NIRS prediction model with a single extraction method for fast screening of the total nutrient contents in spent growing media. The single extraction method used was the AmAc extraction. The novelty of this study is related to four aspects:

- Quantification of nutrients in leaves: most studies focus on assessment of N, but here macro-nutrients other than N were also tested
- Testing the use of NIRS for growing media blends besides peat-based blends, for virgin materials and for spent growing media
- Combination of the application of NIRS in different compartments in controlled environment agriculture, i.e., on the crop (i.e., leaves) versus the horticultural substrate (composts, plant fibers and spent growing media) based on extended datasets in terms of number of samples (>350 leaf samples and >500 substrate samples), matrices and coverage
- Comparison of fast screening for nutrient contents with a single extraction (AmAc) versus NIRS

## 2 Methods

### 2.1 Dataset of strawberry leaves for NIRS

The nutrient content as measured by chemical analysis (see below) in a dataset with 294 strawberry leaves was calibrated using NIRS, and another dataset with 75 leaf samples was used to validate the prediction of the total nutrient content. The leaf samples in both datasets originated from 12 trials with cultivar ‘Elsanta’ grown under variable conditions and experimental setups in different greenhouses, with different growing media blends and a range of fertilizer types and doses. In some of these trials, nutrients supplied by the fertilizers were the limiting factor, while in other trials the supply of nutrients did not limit plant development (e.g., in the case of standard drip fertigation). The samples for both datasets were randomly selected, with samples from each trial included in both the calibration and validation dataset. “Leaf” is defined as the aboveground vegetative biomass, including the stalks and the three separate leaflets of the compound leaves. Remaining fruits were removed before sampling, then the vegetative aboveground biomass (without remaining fruits) was harvested, weighed, manually cut into 2 cm pieces and a subsample was dried at 70°C for further processing. Assessment of optimal range and nutrient deficiency for foliar composition is based on Pritts et al. (2015).

### 2.2 Growing media dataset for NIRS

A dataset of 458 samples of different types of materials used in growing media (calibration dataset) was validated with a dataset of 109 samples (validation dataset). The calibration dataset consisted of 162 compost samples, 202 samples of plant fibers and 94 spent growing media samples. The validation dataset consisted of 39 compost samples, 46 samples of plant fibers and 24 spent growing media samples. The calibration and validation datasets reflected a range of materials, feedstock mixtures and process conditions. The compost samples covered a wide range of compost facilities, seasons of composting, and compost types (i.e., green compost, VFG compost, woody composts). The dataset of plant fibers consisted of different batches of peat, coir products, wood fiber, bark, other woody materials, straw fibers (including different batches of reed or miscanthus straw and flax shives) and plant fibers from nature conservation or landscape management. Spent growing media were collected from different crops and from different growers or trials, and with variable initial composition of the blend. The samples for both datasets were selected at random.

### 2.3 Processing NIRS spectra for leaves and growing media samples

Dry and ground (< 1 mm) leaf or growing media samples were scanned with a FOSS XDS monochromator instrument (FOSS, Hillerød, Denmark) using ISIscan v2.85.3 software. The inverse reflectance (log (1/R)) was measured from 400 to 2500 nm in steps of 0.5 nm. The samples were scanned in duplicate and the spectra were averaged. Data processing and calibration development was executed with WINSI v4.9.0 software. The wavelength range used during scanning of the samples was also the range used for data analysis (400-2500 nm). Depending on the measured characteristic, one or more samples identified as spectral outlier (mahalanobis distance > 3) were removed from the calibration dataset by the software in the first round; additional spectral outliers, if any, were not removed in the second round. To reduce scatter effects, standard normal variate and detrend scatter correction was applied to the spectra. Modified partial least squares regression (Shenk and Westerhaus, 1991) served as calibration model. Cross-validation (by groups, with cycling group formation) was used to select the optimum number of partial least squares (PLS) terms. A second derivative mathematical treatment was used, denoted below as the 2,8,6,1 model with 2: Derivate, second derivate is used, 8: Gap: gap over which the derivate is calculated (range between 4 and 20 nm, here 8 nm), 6: Smooth: smoothing segment of points (4: small amount of smoothing, 20: large amount of smoothing, here 6), and 1: Second smoothing segment: value “1” indicates that no second smooth is used. The standard error of calibration (SEC), standard error of cross validation (SECV) and the determination coefficient (R^2^) of the simple linear regression between reference values (chemical analyses) and NIRS predicted values of the calibration set or validation dataset were calculated.

For the validation set, the standard error of prediction (SEP) and the R^2^ of the linear regression between reference values (chemical analyses) and NIRS predicted values were calculated on the basis of the calibration equations derived from the calibration database. The ratio of Prediction to Deviation, i.e., the ratio of the standard deviation of the validation dataset to the standard error of prediction (SD/SEP) was calculated to reflect the performance of the calibrations.

Outliers were not removed from the validation dataset for the strawberry leaves. Outliers detected in the growing media dataset during the first validation were checked case by case and were excluded from analysis in the validation statistics. After removal of these outliers from the validation dataset, the validation was repeated; no outliers were removed in the second round.

### 2.4 NIRS for fresh and dried (not ground) growing media

A total of 72 samples of fresh and dried composts, plant fibers and spent growing media were collected to test whether the sample processing time could be reduced by eliminating the need to dry and grind the samples. The fresh samples were scanned with a FOSS DS2500 monochromator instrument (FOSS, Hillerød, Denmark) as this equipment allows to scan larger sample volumes (250 ml cup). The inverse reflectance (log (1/R)) was measured from 400 to 2500 nm in steps of 0.5 nm. After scanning, the samples were dried for 48h at 70°C in a ventilated oven and then scanned again.

### 2.5 Fast screening for nutrients with a single extraction

For assessing the prediction of total P, K, Ca and Mg contents based on the AmAc extraction, a dataset with 150 samples of different types of materials used in growing media (calibration dataset) was validated with a dataset with 29 samples (validation dataset). The calibration dataset consisted of 45 compost samples, 36 samples of plant fibers and 69 spent growing media samples, and the validation dataset consisted of 9 compost samples, 7 samples of plant fibers and 13 spent growing media samples. The calibration and validation datasets reflected a range of materials, feedstock mixtures and process conditions. The samples for both datasets were selected at random. The AmAc extraction is executed on a volume basis and the results are reported as g/L substrate. Total contents and AmAc extractable concentrations for P, K, Mg and Ca were therefore both expressed in g/L substrate. Total contents were converted from g/kg DM to g/L substrate based on the dry bulk density. To meet the criterion of a normal distribution, total contents and AmAc extractable concentrations were square root-transformed prior to linear regression analysis. Total contents of the samples in the validation dataset were predicted based on the AmAc extractable concentrations of these samples and the equation of the calibration curve. The predicted total contents for the samples in the validation dataset were then compared with the measured total contents, and the quality of the prediction was assessed based on the R^2^ and the slope of this linear regression model.

### 2.6 Chemical composition of plants and growing media

Methods are based on European Standards developed by CEN, the European Committee for Standardization. European Standard EN numbers refer to the specific standards. Sample preparation of growing media for determination of total nutrient content, dry matter content, moisture content and laboratory compacted bulk density was executed according to EN 13040 (CEN, 2007). Compacted dry bulk density was calculated based on the fresh bulk density and the moisture content of the sample. Leaves and spent growing media were dried at 70°C and mechanically ground with a cutting mill (Pulverisette 19, Fritsch, Idar-Oberstein, Germany) for leaves and in a cross beater mill (SK100, Retsch, Haan, Germany) equipped with heavy-metal-free grinding tools and a 1 mm sieve used for growing media. Samples were stored in closed PP containers before chemical and NIRS analysis. Total N content (determined according to the Dumas method, EN13654-2 (CEN, 2001)) was measured using a Skalar Primacs SNC 100-IC analyzer (Skalar, Breda, The Netherlands). Total contents of P, K, Mg and Ca were determined by 5110 VDV Agilent ICP-OES (Agilent, Santa Clara, CA, USA) in the extract following digestion (120 min at 105°C) of 0.5g dried and ground material with 4 mL HNO_3_ (p.a. 65%) and 12 mL HCl (p.a. 37%) using a DigiPREP MS 200 Block Digestion System (SCP SCIENCE, Québec, Canada). Determination of OM content was done according to EN 13039 (CEN, 2011c) by ashing in a Heraeus muffle oven at 550°C. Electrical conductivity (EC) (EN 13038 (CEN, 2011b)) and pH-H2O (EN 13037 (CEN, 2011a)) were measured on fresh samples in a 1:5 soil to water (v/v) suspension. AmAc extractable K, Ca, P, and Mg concentrations were measured by ICP-OES after extracting the fresh sample in AmAc buffered at pH 4.65 (1:5 solid:water v/v).

### 2.7 Accuracy and precision of predictions based on NIRS or the ammonium acetate extraction

The accuracy and precision of the NIRS predictions and predictions based on the AmAc extraction were assessed based on the slope and the determination coefficient (R^2^) of the validation results, respectively. Accuracy refers to how close a measurement is to the true or accepted value. In this study, the contents measured in the lab are used as the accepted values. The closer the slope of the linear regression of the validation between predicted and measured values is to 1, the higher the accuracy of the prediction. Precision refers to how close measurements of the same item are to each other. The closer the determination coefficient (R^2^) of the linear regression of the validation is to 1, the greater the precision of the prediction.

## 3 Results

### 3.1 Chemical characteristics of leaves, composts, plant fibers and spent growing media

**Figure 1:**
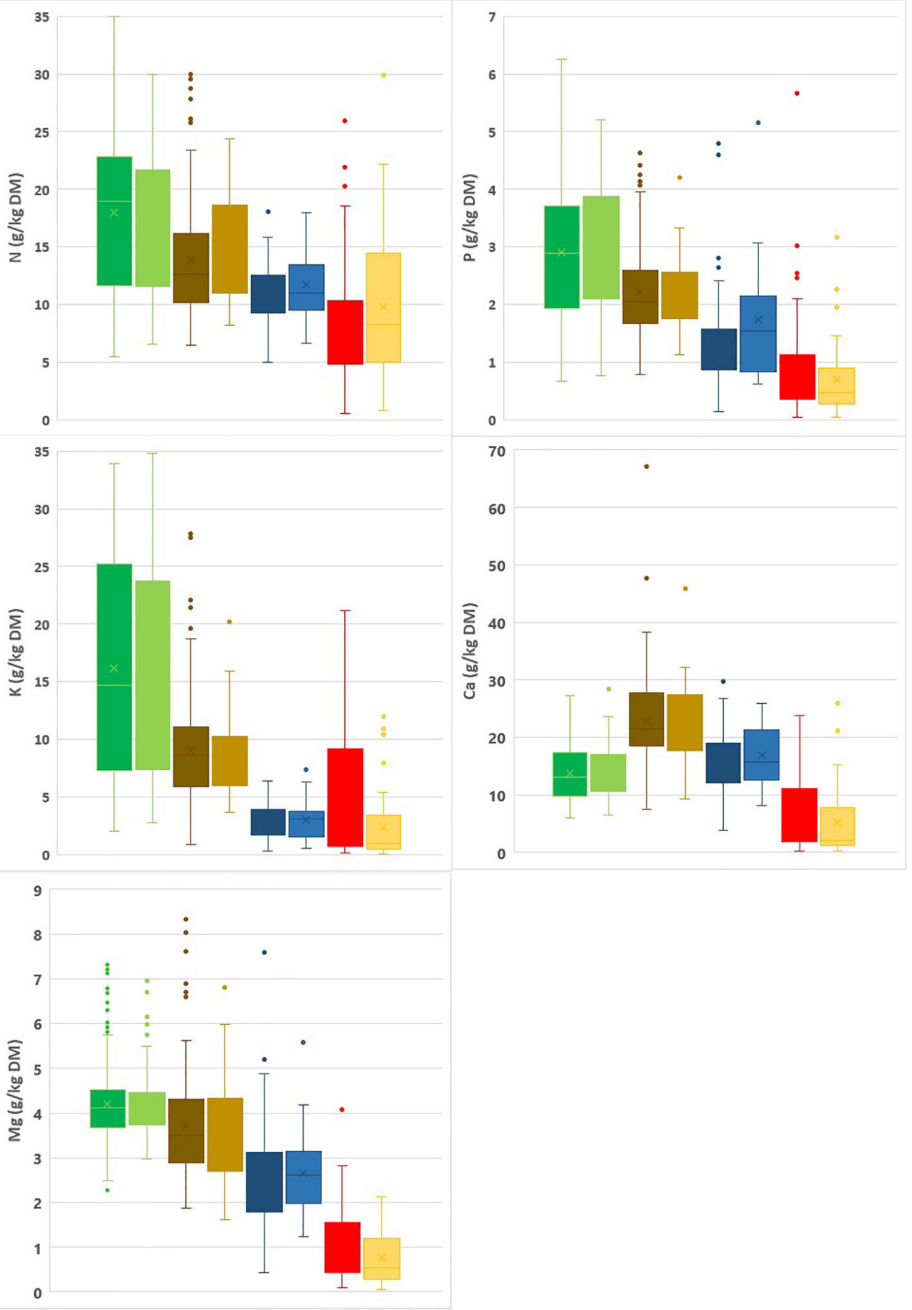
Box plots representing the range in N, P, K, Mg and Ca contents in the calibration (dark) vs validation (light) datasets for leaves (green), composts (brown), spent growing media (blue) and plant fibers (red). A box plot illustrates the variation in samples of a statistical population through their quartiles (the lower and upper box dimension indicates the 25^th^ and 75^th^ percentile) and the lines extending from the box indicating variability outside the upper and lower quartiles. Points indicate outliers.

**Figure 2:**
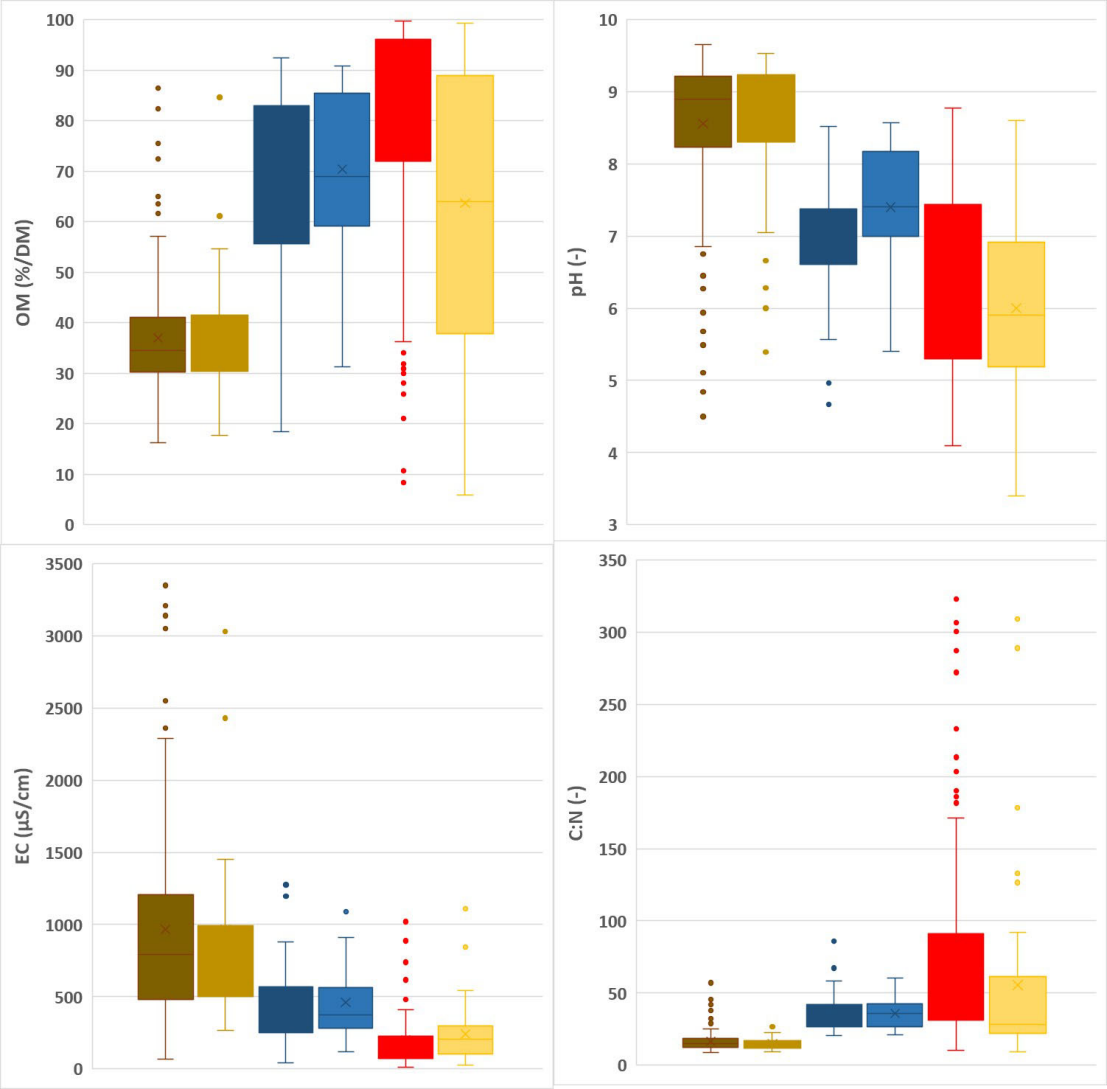
Other chemical characteristics: Box plots representing the range in organic matter (OM), pH, electrical conductivity (EC), C/N, C/P in the calibration (dark) vs validation (light) datasets for composts (brown), spent growing media (blue) and plant fibers (red).

Summary statistics for the calibration and validation datasets are given in **Figures 1**, **2**. N, P and Mg contents are highest in the leaves, lower for spent growing media than for composts, and lowest for the plant fibers. K contents are higher in leaves and composts than in spent growing media and plant fibers. For Ca, composts have the highest contents and plant fibers have the lowest contents. In general, spent growing media and plant fibers were characterized by higher OM, C/N and C/P ratios, and lower pH-H_2_O and EC than for the composts. In cases where some parameters are in the low range within the calibration dataset, prediction by NIRS may be less accurate. There was no risk for a limited range for any of the tested characteristics in the calibration dataset. By combining composts, plant fibers and spent growing media in one dataset, a higher range for each characteristic is obtained. The box plots indicate that the calibration and validation datasets have a similar distribution of measured values. The high range observed in the box plots for each matrix indicates the relevance for assessing the nutrient content and other characteristics of these materials.

### 3.2 NIRS prediction of nutrient contents in leaves

The leaf samples for the calibration dataset were collected in different trials using different fertilizer application systems. In some of these trials, nutrients supplied by the fertilizers were the limiting factor, while in other trials the supply of nutrients did not limit plant development. As a result, the database covered a broad range of nutrient contents in the leaves, with a range of 6-35 g/kg DM, 0.7-6.3 g P/kg DM and 2-29 g K/kg DM. Correlation between nutrient contents in the leaves was highest (R = 0.80) between P and K, while the lowest correlation (R = -0.22) was observed between Mg and Ca. The correlation coefficients are below 0.90, which does not reduce the use of NIRS for fast screening of nutrients. The higher the R² and the lower the SECV (standard error of cross validation), the better the calibration. The best results for strawberry leaves (**Table 1**) in the validation dataset were obtained for total N, P, K and Ca (R^2^ > 0.90) while prediction was somewhat weaker for Mg (R^2^ = 0.86). Similar conclusions can be drawn for the ratio SD/SEP, which was higher than 5 for N and K, higher than 3 for P and Ca, and higher than 2 for Mg (**Table 1**). The higher the ratio SD/SEP, the better the prediction potential, indicating a higher range in the dataset versus a low standard error on the prediction. The slope of the linear regression between predicted values and the results of the chemical analysis is close to 1, with values between 0.97 and 1.05. Only a limited number of outliers were detected in the validation dataset, with a small impact on the validation outcome. These outliers were not removed from the validation dataset.

**Table 1:** Calibration and validation results for total nutrient content in strawberry leaves, mean, standard deviation (SD), SEP and SECV are expressed in %/DM for N or mg/kg DM for the other elements (N: number of samples used for the calibration or validation, DM, dry matter; SEP, standard error of prediction; SECV, standard error of cross validation; R^2^, determination coefficient of the calibration or validation).

		Calibration						Validation								
Constituent		N	Mean	SD	R²	SECV	SD/SECV	N	Mean	SD	SEP	Bias	SEP(C)	Slope	R²	SD/SEP
Total N	%/DM	288	1.8	0.6	0.99	0.1	7.6	75	1.8	0.5	0.1	0.1	0.1	0.99	0.98	6.5
Total P	mg/kg DM	287	2890	1111	0.93	334	3.3	75	2979	1046	340	33	340	0.97	0.90	3.1
Total K	mg/kg DM	286	16054	9560	0.99	1428	6.7	75	16498	9394	1335	180	1332	0.98	0.98	7.0
Total Mg	mg/kg DM	290	4203	837	0.91	303	2.8	75	4229	821	307	-9	309	1.05	0.86	2.7
Total Ca	mg/kg DM	285	13591	4626	0.97	915	5.1	75	14103	4321	1117	162	1113	1.04	0.94	3.9

R^2^ and slope of the validation refer to the linear regression between the total nutrient content predicted based on the calibration equation versus the total nutrient content measured by analysis.

### 3.3 NIRS prediction of nutrient contents and other characteristics in growing media

In contrast to the leaf samples, the outliers in the growing media dataset had a clear impact on the validation outcome, and the outliers detected during the first validation were removed from the validation dataset. Outliers can occur for several reasons: analytical errors, variability of lab analyses or measurements beyond the optimal range of the applied analytical method, or a less accurate prediction of the characteristics of the sample by NIRS. Analytical measurement of very low values may be less correct when they are in the range of the limit of detection or the limit of quantification of the method. High values may suffer from overestimation due to the need for stronger dilution of the extract before analysis, and the related higher analytical uncertainty. The outliers were checked case by case. Outliers were mostly detected at the model boundaries or at the boundaries of the extraction method. In this case, the qualitative categorization of the characteristic as very low or very high may already provide sufficient information. Most outliers could not directly be related to the reliability of the NIRS prediction, but mostly pointed to analytical errors, measurement uncertainty or variability of lab analyses. Outliers for K, Mg and Ca were all compost samples with high contents for these elements. Outliers for EC were also compost samples. EC is the only characteristic that may be prone to an effect of drying of the sample. EC is measured on fresh material, but dried samples were scanned for NIRS. The drying process may have resulted in deviant values.

Correlation between nutrient contents in the growing media was highest (R = 0.88) between Mg and Ca, while the lowest correlation (R = 0.49) was observed between Mg and K, and between N and K. The correlation coefficients are below 0.90, which confirms that NIRS can be used for fast screening of nutrients. The NIRS validation results for assessing chemical properties of materials used in growing media are given in **Table 2**. None of the nutrients in the materials resulted in an R^2^ higher than 0.90. For P, Mg and Ca R^2^ was higher than 0.80, while R^2^ was 0.79 and 0.73 for N and K, respectively. For chemical composition, R^2^ was higher than 0.90 for OM and higher than 0.80 for C/N and pH. The validation for C/P (0.79) and EC (0.67) resulted in a lower R^2^. In general, the values for the ratio SD/SEP were low (= below 3, except for OM) (3.5). In summary, there is potential for indicative prediction of these characteristics in compost, plant fibers and spent growing media. The slope ranges between 0.86 and 1.04 with only a lower value for EC (0.80). The best values for the slope are obtained with the validation for OS, N, pH, C:N, P and Ca (values between 0.96 and 1.04).

**Table 2:** Calibration and validation results for the materials used in growing media.

		Calibration						Validation							
Constituent	Unit	N	Mean	SD	R²	SECV	SD/SECV	N	Mean	SD	SEP	Bias	Slope	R²	SD/SEP
Total N	%/DM	419	1.042	0.468	0.85	0.211	2.2	102	1.195	0.575	0.267	0.051	1.04	0.79	2.2
Total P	mg/kg DM	327	1508	903	0.88	405	2.2	92	1426	962	406	-26	0.96	0.83	2.4
Total K	mg/kg DM	309	5892	4349	0.86	1870	2.3	89	4293	3677	2044	-528	0.86	0.73	1.8
Total Mg	mg/kg DM	306	2444	1359	0.84	603	2.3	88	2000	1341	590	-39	0.89	0.82	2.3
Total Ca	mg/kg DM	312	15500	8501	0.84	3782	2.2	91	13910	9648	3780	548	0.96	0.85	2.6
OM	%/DM	434	61.9	26.4	0.95	6.7	4.0	104	55.4	24.3	6.933	-0.133	0.98	0.92	3.5
pH-H2O	(-)	317	7.00	1.38	0.88	0.53	2.6	86	6.77	1.53	0.59	0.05	1.00	0.85	2.6
EC	µS/cm	310	526	509	0.87	210	2.4	81	453	325	199	-23	0.80	0.67	1.6
C/N	(-)	426	43.0	41.7	0.87	15.4	2.7	103	31.2	26.0	9.3	-2.1	1.02	0.88	2.8
C/P	(-)	321	312	265	0.84	122	2.2	92	345	282	131	-1.4	0.94	0.79	2.2

Mean, standard deviation (SD), SEP and SECV are expressed in the unit for the characteristic (N: number of samples used for the calibration or validation, SEP, standard error of prediction; SECV, standard error of cross validation; R^2^, determination coefficient of the calibration or validation; OM, organic matter; DM, dry matter; EC, electrical conductivity). R^2^ and slope of the validation refer to the linear regression between the value predicted based on the calibration equation versus the value measured by analysis.

### 3.4 NIRS on unprocessed growing media samples

R^2^ values for the calibration curve based on the dried (not ground) samples were comparable to the calibration curve based on the fresh samples (**Table 3**). For the calibration curve based on the dried samples, R^2^ was higher than 0.90 for OM, EC, total P and K. For pH, R^2^ was higher than 0.85, while R^2^ was 0.83 and 0.65 for C:N and total N, respectively. These data indicate that grinding of the samples is not required, resulting in a shorter sample processing time. Further reducing the processing time by scanning fresh rather than dried samples has potential for using NIRS for fast screening of growing media.

**Table 3:** Calibration results for the dried but unprocessed versus fresh materials used in growing media.

		Dried but unprocessed samples						Fresh samples					
Constituent	Unit	N	Mean	SD	R^2^	SECV	SD/SECV	N	Mean	SD	R^2^	SECV	SD/SECV
Total N	%/DM	69	1.5	0.7	0.65	0.5	1.4	69	1.6	0.7	0.87	0.5	1.3
Total P	mg/kg DM	60	1827	1172	0.96	510	2.3	54	1778	1151	0.82	653	1.8
Total K	mg/kg DM	55	6792	4922	0.92	1933	2.5	53	6702	4741	0.95	1946	2.4
OM	%/DM	70	58	25	0.97	8	3	70	57	24	0.96	11	2.1
pH-H2O	(-)	69	6.9	1.5	0.88	0.7	2.1	67	6.9	1.5	0.87	0.8	2.0
EC	µS/cm	69	720	707	0.91	314	2.2	69	766	763	0.91	343	2.2
C/N	(-)	69	32	36	0.83	20	2	68	30	35	0.82	20	1.7

Mean, standard deviation (SD) and standard error of cross validation (SECV) are expressed in the unit for the characteristic (N: number of samples used for the calibration or validation, R^2^: determination coefficient of the calibration, OM, organic matter; DM, dry matter; EC, electrical conductivity).

### 3.5 Single extraction for fast screening

The validation results indicate very good prediction of total P and K using the AmAc extraction, while the results for Ca and Mg indicate good prediction (**Table 4**). Based on the slope of the linear regression equation (**Table 4**), the availability of elements in the AmAc extraction relative to the total contents decreases from K > P > Ca > Mg. The single extraction is an alternative to NIRS for assessing total P, K, Ca and Mg content in growing media.

**Table 4:** Calibration and validation results for assessing total contents based on AmAc extractable concentrations (both are expressed in mg/L substrate).

	Calibration equation	R² Calibration	R² Validation	Slope Validation
P	Sqrt (total P) = 1.22* Sqrt (P-AmAc) + 2.23	0.91	0.90	0.85
K	Sqrt (total K) = 0.93* Sqrt (K-AmAc) + 1.80	0.97	0.98	0.95
Ca	Sqrt (total Ca) = 1.45* Sqrt (Ca-AmAc) - 8.60	0.90	0.87	0.86
Mg	Sqrt (total Mg) = 1.53* Sqrt (Mg-AmAc) - 4.40	0.88	0.88	0.74

R^2^ and slope of the validation refer to the linear regression between the total nutrient content predicted based on the calibration equation versus the measured total nutrient content.

## 4 Discussion

### 4.1 Assessment of nutrient retention in leaves and spent growing media

At the end of greenhouse cultivation based on closed fertigation, nutrients accumulate in both the aboveground plant biomass and the spent growing media. Nutrient contents in leaves may indicate which elements were deficient and thus limited growth and yield. Avoidance of excessive nutrient accumulation at the end of cultivation by optimizing the fertilizer application is a major topic in the transition towards circular horticulture (Vandecasteele et al., 2023). Data on the amounts of nutrients removed by plants and spent media in relation to other nutrient flows in greenhouses are needed to assess the importance of these nutrient pools, and the impact of measures to reduce the risk that they become nutrient sinks (van Tuyll et al., 2022). In conclusion, NIRS as a high throughput technique has a variety of potential applications in controlled environment agriculture: to avoid nutrient limitations or imbalances, to prevent excessive nutrient accumulation, to optimize fertilizer application, to optimize reuse of growing media, or to serve as a benchmarking tool to enable growers to make peer comparisons. The fast and cheap screening using NIRS enables the comparison of nutrient contents in the leaves and spent growing media at the end of cultivation between different years/crop cycles and between greenhouses. This provides useful information about the impact of crop management on nutrient export. The range in N, P, K, Ca and Mg contents in the dataset with strawberry leaves was high. Values for N, P, K, Ca and Mg in the dataset with strawberry leaves were both higher and lower than the nutrient contents for optimal strawberry growth (Pritts et al., 2015). This indicates the potential to further optimize fertigation management in soilless strawberry cultivation in order to avoid suboptimal uptake, which could potentially result in suboptimal yield, as well as luxury consumption and nutrient imbalances, which could potentially result in imbalances with other nutrients and can lead to excessive nutrient exports at crop termination.

Results illustrate that NIRS is a promising tool for predicting the total N, P, K, Ca, and Mg contents in strawberry leaves. This validation is based on one cultivar only and may thus need further validation for other cultivars. The range of nutrients measured in both the dataset of strawberry leaves and the dataset of composts, plant fibers and spent growing media versus the optimum range illustrate the presence of both very high and very low nutrient contents. Correct assessment of these pools can support proper greenhouse management and indicate how these nutrients can be reused or recycled. Nutrients retained in spent growing media may be applied as fertilizer during reuse of spent growing media (Vandecasteele et al., 2020) or during use as soil improver (Vollmer et al., 2022). New materials in growing media like compost or biochar have higher nutrient contents than conventional peat or coir, and can thus potentially replace fertilizers in soilless cultivation systems (Atzori et al., 2021). NIRS, when used as a technique for assessing total nutrient contents in materials used in growing media or in reused spent growing media, may allow for the quantification of the fertilizer replacement value of growing media blends at the start of cultivation.

### 4.2 Leaves versus spent growing media

NIRS is a good technique to assess organic compounds in crops or other materials. The validation curves indicate a better prediction for nutrients in the leaves versus nutrients in the spent growing media. This may indicate that nutrients in the leaves are more bound in organic structures than is the case for spent growing media, but leaf samples are also more homogeneous than growing media samples. Residual nutrients in the root biomass in spent growing media are organically bound, but may represent only a small part of the total nutrient content in the spent growing media. Nutrient retention in spent growing media is mainly due to accumulation of nutrients provided as fertilizer. For plant fibers, nutrients should be included in the plant biomass as organically bound components, comparable to strawberry leaves. This may be valid for compost as it is produced from plant material, but the plant material in compost has undergone a thermophilic processing step. The dataset of composts, plant fibers and spent growing media represents more variable conditions than the dataset with strawberry leaves. The leaf samples in both the calibration and validation dataset were all from one cultivar, while the datasets of spent growing media, composts and plant fibers covered a wide range of different materials from different origins, with the spent growing media covering a wide range of crops. The collected samples for spent growing media, composts and plant fibers thus cover a broader range of materials, processing conditions and represent an overall higher variability. During implementation of the calibration curves for leaves and spent growing media, the calibration datasets must be continually updated with new samples to assure the ongoing reliability of the prediction (in other words, to guarantee that it is sufficiently representative for a wide range of samples).

One calibration curve based on different matrices was used for assessing materials used in growing media (composts and plant fibers), characterization of virgin growing media blends, and characterization of spent growing media as such or processed by composting. This approach results in a calibration curve with a higher range of values for the measured characteristic. It has two main advantages: 1) it is not necessary to categorize an unknown sample as a compost, a plant fiber or a spent growing medium; and 2) the calibration curve is more robust in the context of circular horticulture. A disadvantage may be that SEC, SECV and SEP may increase when datasets are combined. In the transition towards circular horticulture, circular use of materials implies that spent growing media may again be applied as growing media, either through direct reuse, heat treatment or composting.

### 4.3 Fresh versus dried and processed samples or single extraction

Using NIRS or a single extraction for characterization of leaves or growing media strongly reduces both the cost and processing time compared to chemical characterization. Drying of samples greatly impacts processing time as it takes at least 48 h. For some applications, drying and/or grinding is not required and fresh material may even result in a better prediction of chemical characteristics of peat and peat-based growing media using NIRS (Ludwig et al., 2006; Terhoeven-Urselmans et al., 2008) or of the stability and maturity of organic wastes using FTIR (Higashikawa et al., 2014). The test with fresh and dried samples of composts, plant fibers and spent growing media indicated the potential for reducing the processing time of the samples by eliminating the need to dry and grind the samples. Using NIRS with unprocessed fresh and dried growing media samples deserves further attention but may result in less accurate predictions than for dried and ground samples. Multiple regression methods for processing NIRS data are available; future research should focus on a comparison of PLS with other processing methods, including deep learning techniques (Zhang et al., 2019; Li et al., 2023).

NIRS was tested on dried and ground strawberry leaf samples under lab conditions, which represents the optimal scenario (Prananto et al., 2020). These authors concluded that dried and ground samples result in a better calibration compared to fresh leaf samples due to the standardization of moisture and particle size, and that NIRS performed better in a laboratory compared to field conditions due to interfering external factors such as moisture, temperature, solar radiation and leaf orientation (Prananto et al., 2020). However, when using NIRS for monitoring crop development and nutrient status, this technique should be applied on fresh leaves in the field or in the greenhouse (e.g., Ulissi et al., 2011). Based on the high quality validation results for most nutrients in our study, NIRS probably has potential for screening or classifying fresh leaves as well (Borraz-Martinez et al., 2019).

NIRS and AmAc extraction were compared for fast screening of total P, K, Ca and Mg contents in composts, plant fibers and spent growing media. Both screening methods performed equally well in the test, with AmAc extraction being faster and easier to perform. The accuracy of the prediction (based on the slope) was better for NIRS, while the precision of the prediction (based on R^2^ values) was better for the AmAc extraction. From the similar correlation between AmAc-extractable and total nutrients, we conclude that AmAc is a proxy for total nutrient content rather than for nutrient availability in composts, plant fibers and spent growing media. Ammonium acetate extractable concentrations were determined in an extraction buffered at pH 4.65. This pH is lower than the pH used in other extraction protocols, which may explain why the AmAc extraction reflects total rather than available nutrient contents.

## Data availability statement

The raw data supporting the conclusions of this article will be made available by the authors, without undue reservation.

## Author contributions

BV: conceptualization, data collection, writing—original draft preparation. CW: conceptualization, data collection, writing—review & editing. Both authors contributed to the article and approved the submitted version.
